# Imaging-based 3D‐printed anatomical models for preoperative planning in hepatopancreatobiliary surgery: a single-center pilot study, cost analysis, and systematic review

**DOI:** 10.1007/s11547-026-02210-3

**Published:** 2026-04-13

**Authors:** Pasquale Avella, Maria Chiara Brunese, Salvatore Spiezia, Giustiniano Inglese, Paolo Bianco, Francesco Stanzione, Giulia Varriano, Salvatore Cappabianca, Fulvio Calise, Aldo Rocca, Luca Brunese

**Affiliations:** 1 Hepatobiliary and Pancreatic Surgery Unit, Pineta Grande Hospital, 81030 Castel Volturno, Caserta, Italy; 2 Department of Medicine and Health Sciences “V. Tiberio”, University of Molise, 86100 Campobasso, Italy; 3 General and Oncological Surgery, Umberto I Mauriziano Hospital, Corso Turati 62, 10128 Turin, Italy; 4 Department of Precision Medicine, University of Campania “L. Vanvitelli”, 80138 Caserta, Italy; 5 General Surgery Unit, Pineta Grande Hospital, 81030 Castel Volturno, Italy

**Keywords:** 3D printing, Hepatopancreatobiliary surgery, Preoperative planning, Anatomical model, Medical education, Surgical simulation

## Abstract

Patient-specific three-dimensional (3D)-printed models are increasingly used to optimize preoperative planning in complex hepatopancreatobiliary (HPB) surgery. This single-center study evaluated their anatomical accuracy, clinical utility, cost efficiency, and educational value for surgical planning and training. Three patients with complex HPB lesions (pancreatic head adenocarcinoma, ampullary carcinoma, and giant hepatic hemangioma) were selected based on lesion size (> 3 cm) and suspected vascular or biliary involvement. Imaging data were segmented to produce multicolored 3D-printed models, which were used for preoperative simulation and surgical team training. An educational study was also conducted: 56 medical students were randomized to traditional learning (control) or to learning with a 3D-printed model (experimental) before completing an anatomy quiz. In parallel, a systematic review of the literature up to October 8, 2025, was conducted to evaluate the current evidence on the impact and applications of 3D printing in HPB surgery. All patient-specific 3D-printed models reproduced each patient’s anatomy, including tumors and their spatial relationships to vasculature and bile ducts. Model production required ~ 33 h on average, and material cost was ~ €55 per model. Students exposed to 3D-printed models scored higher on anatomy quizzes, indicating significantly better 3D spatial understanding than controls (*p* < 0.001). The systematic review identified 14 studies (2014–2024; 218 patients), confirming that 3D printing improves anatomical understanding, surgical planning, and education while remaining cost-effective. Patient-specific 3D-printed models enhance surgeons’ understanding of complex anatomy, enable personalized operative planning, and advance surgical education in HPB surgery, offering a cost-effective, high-fidelity tool with promising clinical and educational impact.

## Introduction

Hepatopancreatobiliary (HPB) anatomy is highly complex and variable, with significant vascular and biliary branching variations that can pose challenges in planning resections of the liver, pancreas, and biliary tree [1–3]. Careful preoperative planning is critical to achieve clear margins while preserving essential vascular inflow/outflow and functional parenchyma [4–6]. Conventional planning relies on two-dimensional (2D) imaging computed tomography (CT), magnetic resonance imaging (MRI), and angiography [2, 7], as well as 3D mental mapping [8, 9], Intra-Operative Ultrasound (IOUS) [8], indocyanine green fluorescence (ICG) [10–13], and virtual three-dimensional (3D) models [14]. Post-processing platforms, Artificial Intelligence (AI), and radiomics have changed the imaging overview, increasing accessibility to multiplanar reconstruction (MPR) and automatic segmentation, which has improved the quality of pre-operative planning and the multidisciplinary discussions with surgeons, oncologists, pathologists, and clinicians [15–19].

In recent years, patient-specific 3D-printed models have been applied in numerous surgical specialties such as orthopedic surgery, neurosurgery, cardiovascular surgery, thoracic surgery and pediatric craniofacial and skull-base surgery [20–24]. In the realm of abdominal surgery, 3D-printed models have been explored for hepatic and pancreatic tumors, but their systematic use in major HPB resections remains limited [2].

Early case studies suggest that 3D models can improve understanding of anatomy and may lead to reduced operative time, less blood loss, and improved resection margins compared to standard planning [24–28]. This may be particularly critical advantage in elderly patients, who often present with increased surgical risks and may benefit from more precise and individualized preoperative planning [29–31]. In addition, 3D printing has clear educational value [32, 33].

In this context, our study aimed to evaluate the application of 3D-printed anatomical models for preoperative planning in complex HPB surgery.

Primary endpoints were to assess the models’ anatomical fidelity and their influence on surgical decision-making and intraoperative course. Secondary endpoints aim to analyze the costs of the in-house 3D printing process to gauge feasibility and to examine the educational impact of such models through a student survey assessing whether access to a 3D model improves understanding of HPB anatomy.

In addition to the prospective single-center analysis, we conducted a systematic review of the literature to comprehensively evaluate the current evidence on the application, impact, and clinical relevance of 3D-printed model in HPB surgery.

## Materials and methods

### Patient selection

We conducted a prospective observational study on 3D model utilization in HPB surgical planning in HPB Surgery Unit of Pineta Grande Hospital, Italy. Patients were recruited between September 2024 and February 2025 after multidisciplinary evaluation.

Inclusion criteria were designed to identify cases of high anatomical complexity where a 3D-printed model could offer clear benefit during surgery. Eligible patients had one or more of the following: a mass > 3 cm in diameter; suspected vascular involvement (e.g., contact with major hepatic or mesenteric vessels); suspected biliary tree involvement; invasion of adjacent organs (duodenum, colon, stomach); or radiologic evidence of regional lymph node metastases. Patients not candidates for curative surgery (unresectable disease or poor performance status), those with significant uncontrolled comorbidities according to American Society of Anaesthesiologists (ASA) score [34], or with distant metastases were excluded. A total of 38 patients were screened, of whom 3 met criteria and were selected for 3D-printed modeling.

All patients preoperatively gave informed consent for use of their imaging data to create anatomical models.

Data collection was performed to evaluate intra- and postoperative outcomes. Postoperative complications were reported according to Clavien-Dindo classification [35].

### Imaging acquisition

All patients underwent a quadri-phasic CT scan acquired with a standard protocol according to Italian Society of Medical and Interventional Radiology (SIRM) acquisition protocol for the study of HPB district [36]

A non-ionic contrast agent was injected at a rate of 3 ml/s through automatic power injector.

The standard to optimize 3D model building required a scanner with a minimum of 64 detectors. The scan data were 120 kVp, 100–470 mA, slice thickness of 0.5–2 mm.

As for previous AI and radiomics studies [37–39], the images were exported into DICOM files and uploaded to an open-source platform allowing manual or semi-automatic segmentation (3D Slicer; Surgical Planning Lab, Brigham and Women’s Hospital, Boston, MA, USA).

#### Preoperative work up and 3D reconstruction, printing, and assembly

Key structures of interest for modeling included the liver, pancreas, tumor lesion, hepatic and portal veins, arteries, biliary ducts, inferior vena cava, aorta, and adjacent organs as needed. Radiologists and surgeons collaboratively identified target structures on the imaging slices.

Detailed 3D anatomical reconstructions were generated from the imaging data using a 3D reconstruction platform (MIC MEDICAL-NOTE S.R.L.), with additional manual refinement to ensure anatomical accuracy and fidelity. Each organ and lesions were segmented as a separate label.

The CT was primarily used for bony structures and arterial anatomy, while MRI helped delineate biliary ducts and soft tissue margins; the two modalities’ segmentations were merged to create a comprehensive model. After segmentation, the 3D surface meshes of each structure were generated and refined (smoothed and decimated for printing efficiency). The digital models were exported in STL file format.

Before printing, STL files were processed with slicing software (PrusaSlicer 2.5, Prusa Research, Prague, Czech Republic) [40]. We scaled the models to true patient dimensions (1:1 scale) and oriented them to minimize support material. The models were printed using a Prusa Mk4S fused filament fabrication 3D printer (Prusa Research) with polylactic acid (PLA) filament. Different colored PLA filaments were assigned to different anatomical components for clarity: for example, tumors were printed in a distinct color (e.g., purple or yellow), vascular structures in red (arteries) and blue (veins), bile ducts in green. Printing was done at 0.2 mm layer height and ~ 15% infill for solid organs, with higher infill or solid walls for thin vessels to ensure integrity. Complex structures (vasculature, biliary tree) were printed as separate pieces to allow clear visualization and then later assembled in the correct spatial orientation relative to the organ.

After printing completion, the components were carefully cleaned of support material and any minor printing artifacts. Assembly of the full model was performed manually. The printed pieces were aligned according to the original anatomy. Alignment was facilitated by small registration features and keys incorporated into the prints. The pieces were then fixed together using stainless steel M3 screws and cyanoacrylate glue at junction points, mounted on a stable base. The result was a life-size, patient-specific 3D model demonstrating the tumor and all relevant anatomy in correct spatial arrangement.

#### Preoperative planning and anatomic fidelity evaluation

Each 3D-printed model was reviewed in detail by the surgical team prior to the operation. The surgeons used the models to simulate the planned resections and strategize key steps. For the pancreatic tumors, the model allowed inspection of the tumor’s proximity to the Superior Mesenteric Artery (SMA) and Superior Mesenteric Vein (SMV), portal vein, and bile duct. Critical anatomical variants identified on the model were underlined and integrated into the surgical plan. The team rehearsed the tumor resection on the model and planned vascular control and parenchymal transection lines accordingly.

During actual surgery, the attending surgeons qualitatively assessed the usefulness and fidelity of the 3D-printed models: after each surgical procedures, surgeons documented whether the model’s information led to any changes in surgical approach (such as extended resection, preservation of a structure, or alternative intraoperative maneuvers) compared to the initial plan based on imaging alone. Furthermore, they also noted any discrepancies between the model and the operative findings. Tumor margin status (R0 vs. R1) was recorded from pathology reports to evaluate the clinical utility of the models.

#### Educational study design

To quantify the educational impact of 3D-printed models, we conducted a randomized controlled survey among medical students. Participants were fourth to sixth-year medical students who had completed foundational anatomy courses and were attending an HPB surgery lecture at University of Molise in May 2025. Participation was voluntary and anonymous, with electronic informed consent obtained. The survey was delivered via an online platform (Google Forms®, Mountain View, CA, USA) with the link and QR code provided during the lecture. The survey design and reporting followed the CHERRIES guidelines for e-surveys [3, 41].

A total of 56 students participated and were randomly assigned in equal numbers (28 each) to two groups:

an experimental group (Group 1), who had the opportunity to observe and handle a 3D-printed liver and pancreas models before and during the survey, and a control group (Group 2) who responded to the survey while viewing only CT and MRI imaging.

The questionnaire consisted of 16 multiple-choice questions, divided into four sections: (1) respondent demographics (year of medical course); (2) “Anatomy Knowledge”—theoretical macroscopic anatomy of the HPB region (5 questions); (3) “Anatomy Practical”—radiologic anatomy/interpretation (5 questions, each with an image such as a CT scan requiring identification of a labeled structure); and (4) “3D Anatomy”—spatial anatomical relationships and topographic understanding (5 questions). These domains were inspired by the validated *Anatomy Competence Score (ACS)* framework [42], which assesses anatomy knowledge in cognitive (theoretical), practical (image interpretation), and 3D spatial aspects. Each question was worth 1 point if answered correctly, yielding a maximum score of 15 points for the knowledge-based sections (the demographic question was not scored), and sub-scores in each domain (0–5 per domain).

We calculated for each participant a total score (0–15). Mean scores were compared between control and experimental groups. An unpaired two-tailed Student’s *t* test was used to assess differences in mean scores, after verifying normal distribution of the data. Domain-specific scores were also compared similarly. A *p* value < 0.05 was considered statistically significant. Statistical analysis was performed using Prism 9 (GraphPad Software, San Diego, CA, USA) [43].

#### Search strategy

Our systematic review included articles published until July 2025, following Preferred Reporting Items for Systematic Reviews and Meta-Analyses (PRISMA) guidelines [44].

We conducted a comprehensive search of the PubMed database to identify original English-language articles reporting clinical experiences on the use of the 3D printing technology in HPB surgery. The search strategy included terms for 3D printing technologies (“3D printing,” “additive manufacturing,” “anatomic models”) combined with liver, biliary, and pancreatic diseases (“liver,” “biliary tract,” “pancreas”) and surgical procedures (“surgery,” “preoperative planning,” “simulation”). Studies primarily focused on bioprinting, tissue engineering, or educational applications were excluded.

An initial screening was performed independently by two authors (P.A., S.S.) based on titles and abstracts. Duplicates were excluded prior to full-text assessment. Subsequently, at least two blinded reviewers (S.S. and G.I.) used Rayyan (Rayyan Systems Inc., Doha, Qatar) to identify and analyze potentially relevant studies. Discrepancies in article selection were resolved by discussion and consensus among all authors. All retrieved references were exported to the EndNote™ 21 reference management software (Clarivate, Philadelphia, PA, USA). The final review and inclusion of eligible studies were completed in August 2025.

#### Inclusion and exclusion criteria

The main study objectives have been formulated according to the Patients, Intervention, Comparison, and Outcomes method [45] (Table 1).

**Table 1:** Patients, Intervention, Comparison, and Outcomes method used to screen experiences in the literature

Population	Adults undergoing complex HPB surgery for hepatic, pancreatic, or biliary tumors with challenging anatomy
Intervention	Patient-specific imaging-based 3D-printed anatomical models for preoperative planning
Comparison	Conventional 2D imaging and virtual 3D visualization without printed models
Outcomes	Anatomical fidelity, impact on surgical planning and intraoperative conduct, and cost efficiency

Only studies that met specific methodological and linguistic requirements were included. Eligible papers were those written in English, conducted on human subjects aged ≥ 18 years, and available in full-text form free of charge. Furthermore, only original research articles reporting clinical, anatomical, or surgical applications of 3D-printed models in HPB surgery were considered for inclusion.

Studies were excluded if they did not fulfill the above-mentioned criteria or if they presented methodological inconsistencies with the scope of the review. In particular, we excluded narrative or systematic reviews, as well as studies describing only virtual or computer-generated 3D reconstructions without the use of physical printed models. Articles focusing primarily on thermoablation techniques or liver transplantation were also omitted. Additionally, studies not fully accessible in complete text and those involving animal experiments were excluded from the final analysis.

#### Data extraction

**Fig. 1:**
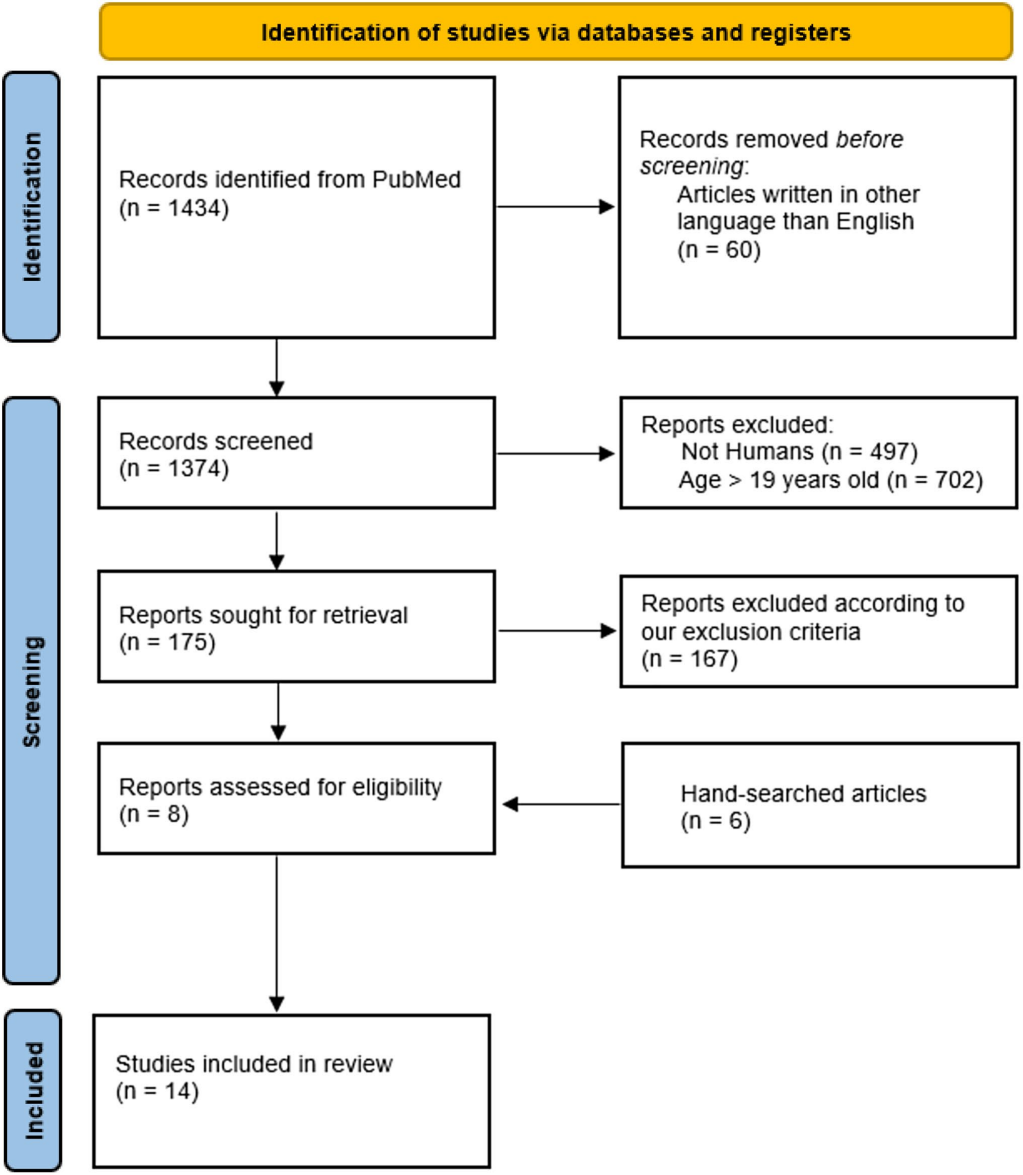
PRISMA flow chart documented the literature review process

Reports were divided into three distinct groups, namely irrelevant, relevant, and unsure. Studies determined to be irrelevant by both reviewers were excluded, while papers that were relevant or unsure by at least one reviewer were analyzed thanks to a full-text evaluation. A PRISMA flow chart documented the entire search and selection process (Fig. 1).

Using a standardized data extraction form, all authors recorded key details for each study, such as the first author, year of publication, country of origin, study design, total number of patients, type of disease, aim of the study, imaging method for 3D reconstruction, relevant finding, 3D Modeling Software/3D-printed Method, 3D-printed Materials/Production Cost and Time.

In the case of missing information, the authors performed calculations based on the available data.

## Results

### Baseline characteristics of patients underwent surgery

Between September 2024 and February 2025, 38 patients affected by HPB diseases were evaluated by our multidisciplinary team. 13 out of 38 (34.21%) were selected for 3D virtual model of anatomy, while 3 out of 38 (7.89%) met inclusion criteria to perform 3D-printed model for surgical planning: (1) pancreatic uncinate process adenocarcinoma (borderline resectable due to proximity to the SMA [46, 47]), (2) ampullary adenocarcinoma, and (3) giant hepatic hemangioma (encasing intrahepatic vascular structures). The baseline characteristics of patients and intra- and postoperative outcomes are reported in Table 2.

**Table 2:** Baseline characteristics, intra- and postoperative outcomes of patients underwent hepatobiliary and pancreatic surgery after 3D-printed model building

Patient	#1	#2	#3
Age, years	85	74	62
BMI, kg/m^2^	28.73	21.91	25.47
CCI	5	4	5
ASA score	III	II	III
Type of tumor	Borderline resectable pancreatic uncinate process adenocarcinoma	Ampullary adenocarcinoma	Giant Liver Hemangioma
Surgical procedure	Total pancreaticoduodenectomy according to Kimura technique	Pancreaticoduodenectomy	Laparoscopic left lateral sectionectomy
Relevant anatomy features	Tumor 170° contact SMA	Dilatation of main pancreatic and common bile ducts	Left gastric artery gives rise to the left hepatic artery
3D-printed model building, hours	40	32	26
Operative time, min	460	360	220
Estimated blood loss, ml	350	430	250
Intraoperative complications, yes/no	No	No	No
PO-ICU, yes/no	Yes	Yes	Yes
CD classification	II	I	I
Length of stay, days	15	12	3
R0 resection, yes/no	Yes	Yes	Yes
30-day mortality, yes/no	No	No	No
90-day mortality, yes/no	No	No	No

*BMI* Body mass index; *CCI* Charlson Comorbidity Index; *ASA* American Society of Anaesthesiologists; *PO-ICU* Post-Operative Intensive Care Unit; *CD* Clavien-Dindo classification; *SMA* superior mesenteric artery

### Model building

The end-to-end production time for each model (from image processing to finished model) averaged around 32.7 hours (h) although much of this time was automated printing (Table 3). Specifically, segmentation and 3D reconstruction required ~ 8 h of specialized work per case, slicing and print preparation ~ 1–2 h, printing itself ~ 23–30 h (machine time), and final assembly ~ 3 h of manual work. These durations varied with the complexity of patient anatomy (larger or more complex models took longer to segment and print). While printing did introduce a time investment, it was feasible to perform image processing and printing in parallel with other preoperative preparations (e.g., printing was done over several nights). All models were ready in time for surgical planning sessions, typically 5–7 days before surgery.

**Table 3:** Segmentation and printing times (in min) for anatomical compartments across three patient-specific 3D models

Patient	#1	#2	#3
Arterial compartment	374 min	265 min	320 min
Venous compartment	518 min	504 min	315 min
Parenchyma and/or lesion	191 min	97 min	575 min
Biliary compartment	358 min	565 min	248 min
Duodenum	158 min	0 min	0 min
Total	1599 min (26 h 39 min)	1431 min (23 h 51 min)	1458 min (24 h 18 min)

The table details the time required for modeling of each anatomical component—arterial, venous, biliary compartments, parenchyma and lesion, and duodenum—for three representative cases: (#1) adenocarcinoma of the pancreatic uncinate process, (#2) adenocarcinoma of the ampulla of Vater, and (#3) giant hepatic hemangioma

**Fig. 2:**
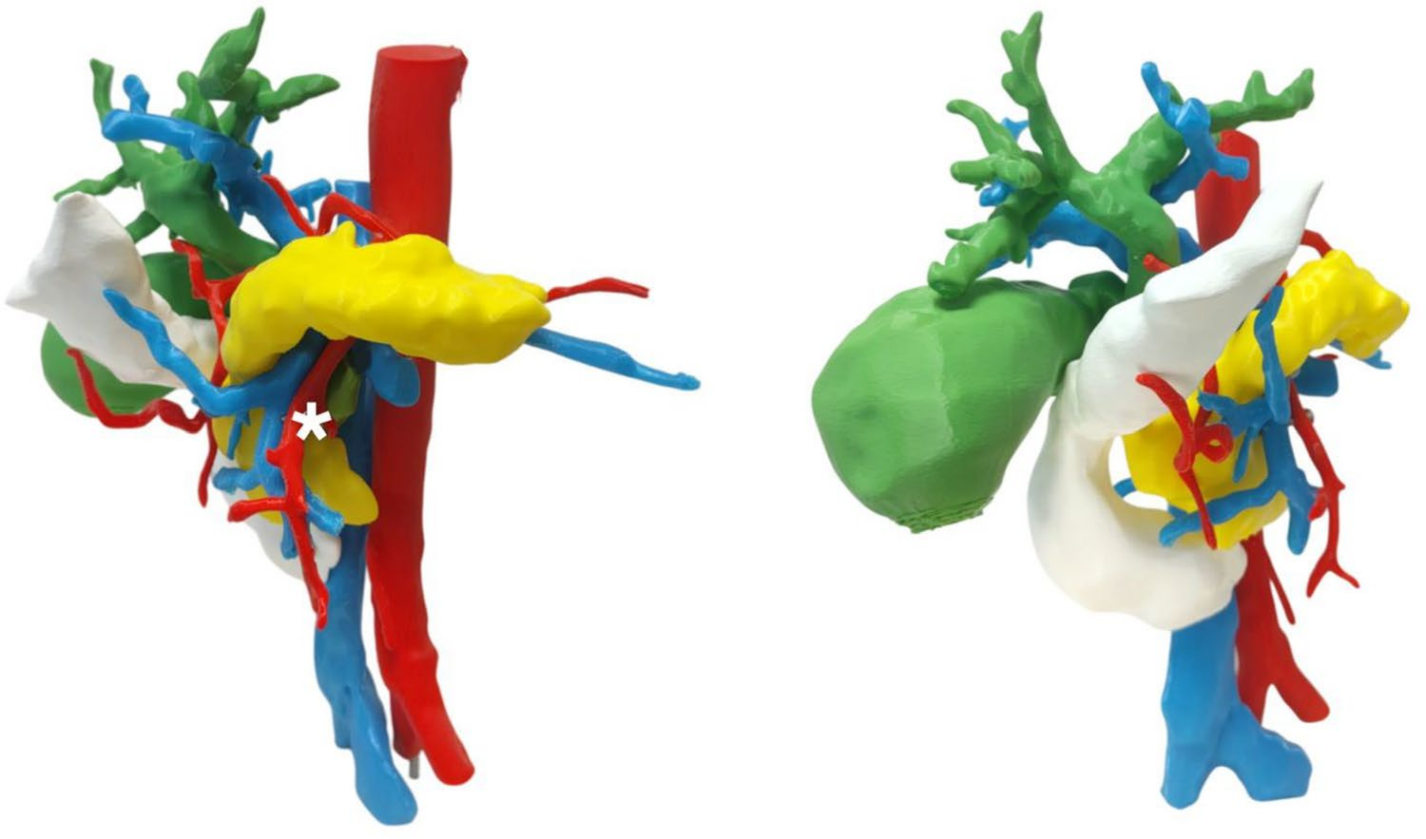
Preoperative 3D reconstruction based on imaging for a patient with a pancreatic uncinate process adenocarcinoma. The 3D-printed model mimics the pancreas (yellow), tumor (lime green), superior mesenteric artery (red) and vein (blue), and liver (green). The 3D model provides an intuitive visualization of the tumor’s encasement of the mesenteric vessels (*), complementing the cross-sectional images

**Fig. 3:**
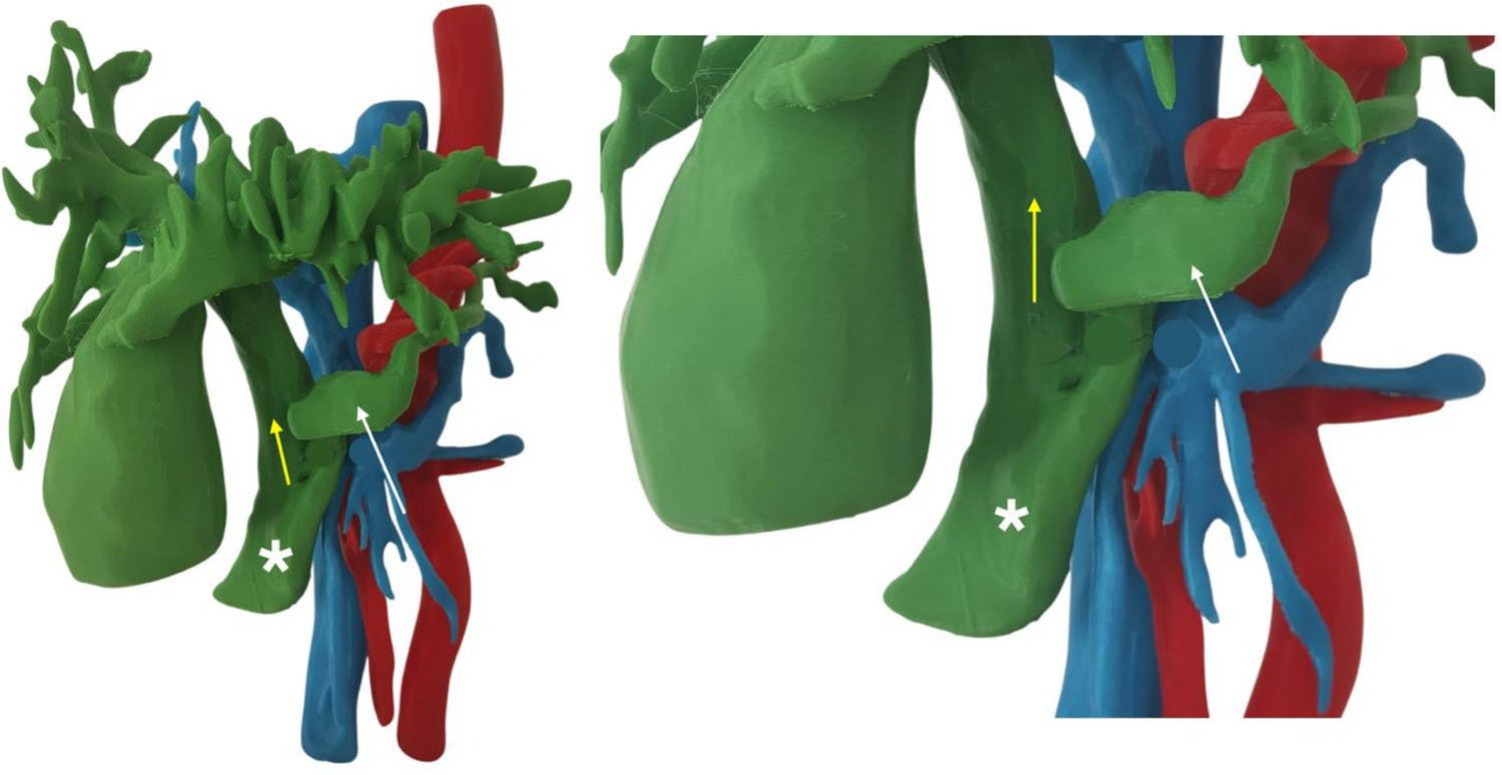
A patient-specific 3D-printed anatomical model used for surgical planning in the case of ampullary adenocarcinoma. The reconstruction delineates the spatial and anatomical relationships between the respective structures. The model depicts in a color-coded way: gallbladder (green), common bile duct (green, yellow arrow), the main pancreatic duct (green, white arrow), the portal vein and its main branches (blue), the aorta (red), the inferior vena cava (blue) and the hepatic artery (red), the ampullary adenocarcinoma (*).

**Fig. 4:**
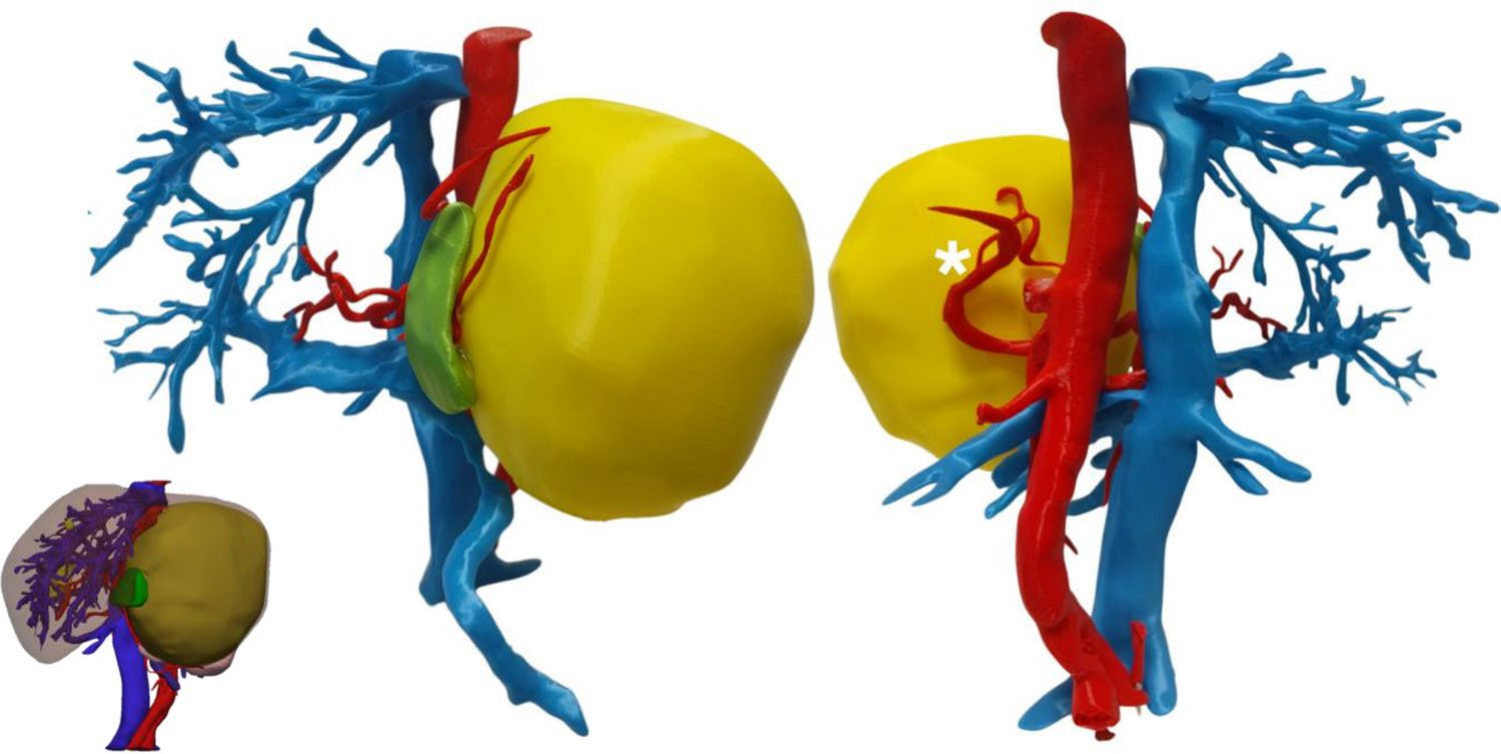
A patient-specific 3D-printed anatomical model used for surgical planning in the case of a giant hepatic hemangioma. On the left side it is shown the three-dimensional anatomical reconstruction of the case, using a 3D reconstruction platform (MIC MEDICAL-NOTE S.R.L.). The model depicts the left lobe of the liver and associated structures. The hemangioma (bright yellow, H) is shown in its actual location within segments II–III. Surrounding anatomy is color-coded: the hepatic artery (red), portal vein branches (blue), hepatic veins (purple), and gallbladder (green) are all represented. The left gastric artery gives rise to the left hepatic artery (*)

Figures 2, 3, and 4 represent the final 3D-printed models.

We also estimated production costs of our cases (Table 4). As detailed in Table 4, the material cost for all three models was approximately €170.78 in total. On a per model basis, this averages to about €55 for materials (PLA, fasteners, etc.). The 3D printer used is an affordable desktop model (about €1000 initial investment) and was reused for all cases; its depreciation or maintenance costs were not significant over the study. Electricity cost for printing was around €12 per model.

**Table 4:** Estimated production costs for the patient-specific 3D models (materials for three models in total). Consumable prices are in Euros

Cost item	Quantity	Unit price, €	Total, €
PLA filament (1 kg spools, various colors)	4 spools (≈1 kg each)	20.00	80.00
Cyanoacrylate glue (industrial grade)	1 bottle	3.99	3.99
Self-tapping screws (M3 × 12 mm)	1 box (≈100 pieces)	5.99	5.99
Wooden base (50 × 50 cm)	3	8.00	24.00
Laser-engraved aluminum label	1	20.00	20.00
3D printer electricity (92 h* at €0.40/kWh)	–	–	36.80
Total for 3 models	–	–	170.78

*Printing time total for all three models

Nevertheless, labor costs are more difficult to quantify: in our experience, surgical research fellows and engineers performed segmentation and assembly as part of a research project. If valued at standard technical staff rates, the ~ 10 h of labor per model would still represent a modest expense relative to typical hospital costs.

### Intraoperative assessment of 3D-printed models

Intraoperatively, the models were validated against real anatomy. Clinically, using the 3D models had a tangible influence on surgical planning and execution: Surgeons reported that the tactile and 1:1 scale 3D-printed models greatly enhanced their spatial understanding of anatomical relationships.

Notably, there were no major discrepancies between model and operative findings—all tumors and their extensions, as well as vascular and biliary anatomies, were accurately depicted.

Minor differences (such as exact degrees of fibrotic adherence of tissues) were inherent to pathology and did not reflect inaccuracies in the model per se.

All resected specimens’ pathology confirmed the imaging dimensions of tumors, further supporting model accuracy. In all three cases, negative resection margins (R0) were achieved, which the surgeons partly attribute to the enhanced planning with the 3D models.

In Patient #1, the model clearly demonstrated the tumor’s 170° with the SMA. With the model in hand, the team planned for a possible vascular resection/reconstruction (Fig. 2). Indeed, during surgery the tumor was found to be abutting the SMA adventitia as predicted. A short segment of the SMA’s adventitial layer was safely peeled and repaired with a primary suture, a maneuver anticipated and practiced on the model. This preparation contributed to smoother intraoperative decision-making and avoidance of an unplanned conversion to a more extensive vascular procedure.

In Patient #2, the 3D-printed model of the pancreas, distal bile duct, and surrounding structures, reinforced the standard Whipple procedure plan (Fig. 3). It did not necessitate a change in surgical approach, but surgeons noted that it served as a valuable reference during lymph node dissection around the interaortocaval and pancreatoduodenal regions, helping to orient the team to the patient’s specific vascular anatomy.

In Patient #3, the model highlighted an accessory left hepatic artery (originating from the left gastric artery) supplying the left lateral liver segments, running close to the hemangioma (Fig. 4). This variant vessel was apparent on imaging but appreciated far more clearly on the 3D model, allowing surgeons to plan its preservation. Indeed, during the bisectionectomy of liver segments 2 and 3, the accessory artery was preserved. The model also helped define resection planes to avoid unnecessary sacrifice of uninvolved liver segments.

### Educational impact

**Fig. 5:**
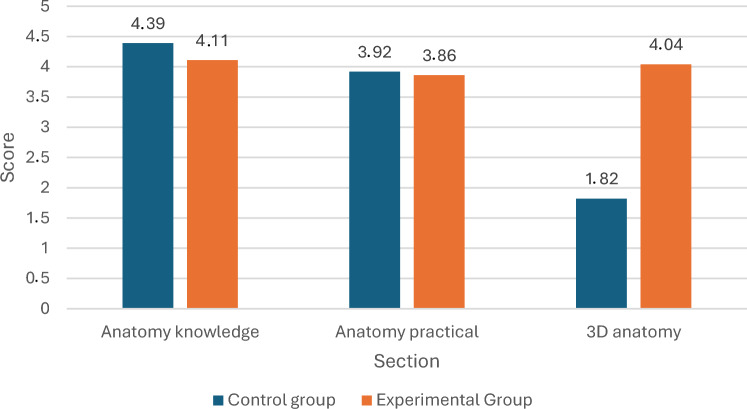
The bar chart compares the performance of a control group and an experimental group across three assessment domains in the field of anatomy: *Anatomy Knowledge*, *Anatomy Practical*, and *3D Anatomy*. While the control group demonstrated slightly better performance in traditional assessments, the experimental group showed a marked advantage in the domain specifically related to 3D anatomical learning

Fifty-six medical students completed the anatomy survey (28 control, 28 experimental). Participant demographics (year of medical study) were balanced between groups; all were senior medical students with prior anatomy coursework. Overall quiz performance was higher in the group that had access to a 3D-printed model. The experimental group’s average total score was 12.0 ± 1.1 out of 15, compared to 10.11 ± 1.3 in the control group (*p* < 0.001). This corresponds to an improvement of roughly 19% in overall score with the use of a 3D model. Figure 5 illustrates the score distributions for the two groups, showing a right-shift for the experimental group’s scores (most experimental students scored in the 11–13 range, versus 9–11 for controls).

In the *Anatomy Knowledge* domain, both groups performed similarly. The control group mean was 4.36 ± 0.83 out of 5, and the experimental group mean was 4.25 ± 0.65 (*p* = 0.583). This suggests that providing a 3D model did not notably change factual recall of anatomy, which is expected as both groups presumably had comparable baseline knowledge from prior study.

Similarly, in the *Anatomy Practical* domain (radiologic anatomy interpretation), the experimental group mean (3.82 ± 0.61) was not significantly different from the control mean (3.93 ± 1.05; *p* = 0.634). Both groups had moderate success in identifying structures on CT/MR images, with the experimental group showing a slight positive trend. These results indicate that while the 3D model primarily enhanced spatial understanding, it did not detract from or add much to general anatomy knowledge or imaging interpretation in the short term.

The most striking difference was observed in the *3D Anatomy domain* (spatial/topographical understanding). The experimental group scored significantly higher in this domain (mean 4.11 ± 0.57 out of 5) than the control group (1.82 ± 1.12 out of 5). This difference was highly significant (*t* =  − 9.64, *p* =  < 0.0001). In fact, nearly all experimental group students answered much of the 3D spatial questions correctly, whereas control students often struggled with these. No student in the control group achieved full marks in the 3D domain, whereas 32% of the experimental group did.

### Systematic review

A total of 1434 articles were initially obtained (Fig. 1). According to exclusion criteria, 1426 studies were excluded. After the addition of 6 hand-searched studies, 14 articles were finally selected [28, 48–60] (Table 5): 7 case report, 1 case series, 1 clinical observational study, 1 prospective observational study, 1 prospective preliminary study, 1 cohort study, 1 prospective comparative study, 1 randomized controlled study. The cumulative study population encompassed 218 patients. The publication period ranges from 2014 to 2024. The PRISMA flow diagram outlining the study selection process is presented in Fig. 1.

**Table 5:** Articles included in the systematic review

First author	Year	Country of origin	Study design	Sample	Disease	Aim	Imaging method	Relevant findings	3D Modeling Software/3DP Method	3DP Materials/ Production Costs and Time
Igami et al. [48]	2014	Japan	Case report	2 patients	CRLM (invisible small tumors)	To evaluate use of a 3D-printed liver for hepatectomy of small tumors invisible to intraoperative ultrasonography	MDCT	3D-printed liver helped localize small, invisible tumors; successful resection with negative margins	Software: PLUTO; method: STL via "Marching Cubes" procedures; printed with AGILISTA-3100	Material: rigid acrylic resin + urethane paint;cost ~ 50,000 JPY (70% scale) and ~ 18 h print;
Igami et al. [49]	2017	Japan	Case report	1 patient	CRLM	Describe application of 3D-printed model for minor hepatectomy after liver partition between anterior and posterior sectors	MDCT	3D model facilitated identification of resection line and precise liver partition landmarks; surgery successful	Software: PLUTO; method: STL; printed with AGILISTA-3100	Material: rigid acrylic resin; cost ~ 50,000 JPY (70% scale) and ~ 18 h print; 110,000 JPY (full size) and ~ 36 h print
Witowski et al. [50]	2017	Poland	Case report (technical report)	1 patient	CRLM	Develop a cost-effective, transparent, patient-specific 3D-printed liver model for preoperative planning before laparoscopic right hemihepatectomy	CT	Transparent, full-sized 3D liver model produced for improved anatomical visualization and surgical planning	Horos, Meshmixer and Blender software program; Open-source Cura software; FDM Ultimaker 2 + 3D printer	Materials: PLA + silicone + XTC-3D resin; cost < $150; printing 72 h, curing 72 h
Joo et al. [51]	2019	South Korea	Prospective preliminary study	20 patients (98 FLLs)	Focal liver lesions (HCC, CRLM, Hepatic metastasis other than CRLM)	To assess usefulness of 3D-printed transparent liver models for lesion-by-lesion imaging-pathologic matching	MRI (Hepatobiliary phase, Gadoxetic acid–enhanced)	Detection rate improved from 82.7% → 99.0% with 3D model; enhanced lesion matching, especially for ≤ 10 mm lesions	Software: MEDIP; method: printed with 3D-printed MakerBot Replicator 2x + silicone casting	Material: ABS + transparent silicone; cost: NA; printing: 50% scale, with 10–30 min analysis per specimen
Muguruza Blanco et al. [52]	2019	Spain	Case report	1 patient	Hepatic metastases	Create a patient-specific 3DPLM featuring functionalized internal surfaces for application in surgical planning	CT and MRI	Translucent and flexible 3DPLMs supported surgical simulation and planning by enabling surgeons to practice incisions while visualizing the spatial relationships among intrahepatic structures	Mimics, Meshmixer, Keyshot FFF	PLA and PVA–3DP mould + 3 silicone rubbers tested ~ €120 per model ~ 10 h for segmentation and rendering time per model
Laureiro et al. [53]	2020	Italy	Case report	1 patient	Klatskin Tumor	Describe the usefulness of a 3DPLM in preoperative planning and intraoperative guidance for a complex liver resection	CT	3DPLM enhanced surgeons’ ability to determine the optimal dissection plane and the most suitable vascular reconstruction approach	3D model software NA; 3DP method NA	3DP materials NA; production cost ~ €1200; production time ~ 35 h
Witowski et al. [54]	2020	Poland	Prospective observational study	19 patients	CRLM, HCC, Solitary Fibrous tumor	Evaluate impact of 3D-printed liver models on decision-making in LLR with intraoperative ultrasound	CT (arterial, portal, delayed phases)	3DP altered surgical plan in 26% of cases; improved spatial understanding; useful adjunct to IOUS. 3DPLMs changed the preoperative surgical plan for several patients by providing better comprehension of spatial relationships between liver lesions and vasculature	Software: 3D Slicer; segmentation: Horos; method: FDM printing + silicone casting	Material: PLA + silicone; preparation time ~ 5 days; PLA:intrahepatic structures; Transparent silicone: parenchyma; production cost: NA; production time: ~ 5 days
Lopez-Lopez et al. [28]	2021	Spain	Case report, descriptive survey, randomized controlled	35 patients	CRLM; ICC, Klastkin tumor, hemangioma, adenoma, focal nodular hyperplasia, HCC, gallbladder cancer, primary sarcoma, sarcoma metastases, adrenal metastases	Validate the accuracy of 3DPLMs and assess their usefulness in surgical planning for complex liver resections, as well as in education and patient information delivery	CT and MRI	3DPLMs facilitated surgical planning and education, enhancing patients’ understanding of their pathology and the planned surgical procedure, though they did not influence the surgical outcome	3D-MSP reconstruction software; 3DP method: NA	TPUR–parenchyma; ABS–intrahepatic structures; production cost €950; production time 22 h
Rhu et al. [55]	2021	Korea	Case report	1 patient	HCC and intrahepatic metastasis	Develop a 3DPLM to support surgical planning and guide the intraoperative procedure for a complex LCR case	MRI	3DPLM enhanced the understanding of tumor location during preoperative planning and increased comprehension of the surgical field during surgery, aiding in the guidance of resection	Mimics FFF	3DP materials NA; $15 material costs; 6 h printing time
Tooulias et al. [56]	2021	Greece	Case report	1 patient	Liver Cancer	Create an accurate, patient-specific 3DPLM that includes a tumor model intended for resection	CT	Use of 3DPLMs may enable more precise tissue resection, helping to preserve a larger amount of healthy parenchyma.	3D model software NA; 3DP method NA	3DP materials NA; production cost NA; production time NA
Cheng et al. [57]	2022	China	Prospective comparative	54 patients	HCC, Hepatolithiasis, Hilar Cholangiocarcinoma	Develop a method to enhance the efficiency and reduce the cost of 3DPLM production, and investigate the value of 3DPLMs in complex laparoscopic hepatectomy	CT	3DPLMs supported surgical planning and intraoperative decision-making; however, no significant differences in patient outcomes were observed between the 3DP and non-3DP groups	E3D, Ultimaker Cura SLA	Photosensitive resin; Mean production cost: $104.40; Mean production time: 56.8 h
Valls-Esteve et al*.* [58]	2023	Spain	Case series	3 patients	Pediatric hepatoblastoma, hepatic hamartoma, biliary rhabdomyosarcoma	Develop low-cost, patient-specific soft 3D models for surgical planning and hands-on training	CT	Transparent, soft silicone ‘parenchyma’ cast inside 3DP mould enhanced surgical planning and allowed physical practice/rehearsal using surgical equipment	IntelliSpace Portal FFF and SLS	Materials: Silicone, PLA moulds; low cost (< 500 €); time 8–24 h per model
Igami et al. [59]	2024	Japan	Clinical observational study	17 patients	HCC and CRLM	Assess clinical value of 3D-printed liver models for navigation during thrice or more repeated hepatectomy using conversation analysis	MDCT	3D models enhanced anatomical understanding and intraoperative decision-making, especially for non-expert surgeons	Software: PLUTO; Method: STL via "Marching Cubes" procedures; printed with AGILISTA-3100	Material: rigid acrylic resin; manually finished with urethane; Cost: NA
Yao et al. [60]	2024	China	Cohort Study	62 patients (31 acquired the guidance of a 3d-printed model)	Intrahepatic cholelithiasis, HCC, ICC)	Clarify the improvement effect of 3D printing models on intraoperative and postoperative complications	CT	3D model was an independent protective factor in decreasing postoperative complications. Subgroup analysis also showed that a 3D model could decrease postoperative complications, especially for bile leakage in patients with intrahepatic cholelithiasis	E3D digital medical modeling software V17.06, Cura 4.4.1 slicing software; printed with SL600 printer	Material: NA; cost: NA; time production: NA

*ABS* acrylonitrile butadiene styrene; AGILISTA-3100 (Keyence Co., Osaka, Japan); Blender (Blender Foundation, Amsterdam, Netherlands; open-source software); *CRLM* colorectal liver metastasis; *CT* computed tomography; Cura (Ultimaker, Geldermalsen, Netherlands); Cura 4.4.1 (an open-source slicing software from Ultimaker in the USA); E3D digital medical modeling software V17.06 (Central and Southern E3D Digital Medical and Virtual Reality Research Center in China); *FDM* Fused Deposition Modeling; *FFF* fused filament fabrication; *FLLs* focal liver lesions; gadoxetic acid (Primovist; Bayer Schering Pharma AG, Berlin, Germany; *HBP* hepatopancreatobiliary; *HCC* hepatocellular carcinoma; *h* hours; Horos (open source; horosproject.org); *ICC* intrahepatic cholangiocarcinoma; IntelliSpace Portal (Philips, The Netherlands); *JPY* Japanese yen; Keyshot (Luxion, Costa Mesa, CA, USA); *LLR* laparoscopic liver resections; MakerBot Replicator 2x (New York, NY); MDCT: multidetector row computed tomography; MEDIP (MEDICALIP, Co, Ltd, Seoul, South Korea); Meshmixer (Autodesk, Inc., San Rafael, CA, USA); Mimics (Materialise, Leuven, Belgium; MRI: magnetic resonance imaging; NA: not available; PLA: polylactic acid; PLUTO (Graduate School of Information Science of Nagoya University, Japan); *PVA* polyvinyl alcohol; *SLA* stereolithography; Slicer (open source, slicer.org); *SLS* selective laser sintering; SL600 printer (ZhongRuiZhiChuang3D Technology Co., LTD. in Suzhou, China); *STL* stereolithography; *TPUR* transparent polyurethane rubber; Ultimaker Cura (Ultimaker, New York, NY, USA); XTC3D (Smooth-On, Inc., Macungie, PA, USA); *3D* three dimensional; 3D-MSP (Cella Medical Solutions, Bajo, Spain); *3DP* 3D printed; 3DPLMs: 3D-printed liver model models; ~ : approximatively; €: euros, $: dollar

The most frequently addressed pathologies included colorectal liver metastases (CRLM) [28, 48–51, 54, 59] and hepatocellular carcinoma (HCC) [28, 51, 54, 55, 57, 59, 60], together accounting for more than half of all reported cases. Other indications comprised intrahepatic cholangiocarcinoma (ICC) [60], intrahepatic cholelithiasis [57, 60], Klatskin tumor [28, 53, 57], and rare pediatric hepatic lesions such as hepatoblastoma, hepatic hamartoma, and biliary rhabdomyosarcoma [58]. One article reported hepatic metastases without specifying the primary tumor of origin [52], while another described a case of liver cancer without further histological characterization [56]. In a separate study, the pathology involved a solitary fibrous tumor of the liver [54], while another paper addressed hepatic metastases originating from a non-colorectal primary malignancy [51]. Furthermore, in one comprehensive series, in addition to CRLM, HCC, and Klatskin tumors, the reported pathologies also encompassed hepatic hemangioma, hepatic adenoma, focal nodular hyperplasia (FNH), gallbladder carcinoma, primary hepatic sarcoma, sarcomatous metastases, and adrenal metastases, reflecting the wide spectrum of hepatobiliary and metastatic diseases addressed through 3D printing applications in surgical planning [28].

Across the included studies, the primary aims showed considerable variability but converged toward a shared objective of evaluating the role of 3D-printed liver models in preoperative surgical planning and intraoperative navigation. Several authors specifically emphasized technical validation and feasibility of model fabrication [48–50, 52, 53, 56, 58, 59], while others sought to assess the clinical impact on surgical decision-making and outcomes, including operative planning modification and complication reduction [28, 51, 54, 57, 60].

Regarding imaging protocols, contrast-enhanced CT was the most widely used modality [28, 48–50, 53, 54, 56–60], while MRI, either alone or combined with CT, was used in either alone or combined with MRI in 4 studies [28, 51, 52, 55].

In terms of findings, nearly all authors reported that 3D-printed models enhanced spatial comprehension of complex hepatic anatomy and improved preoperative visualization of tumor–vessel relationships. Quantitatively, Witowski et al. [54] documented a 26% rate of surgical plan modifications following model review, while Joo et al. [51] observed a marked increase in lesion detection accuracy (from 82.7 to 99.0%). Similarly, Yao et al. [60] identified 3D modeling as an independent protective factor for postoperative complications, particularly bile leakage in patients with intrahepatic cholelithiasis. Lopez-Lopez et al. [28] underscored the educational and communicative value of 3D models in enhancing patient understanding and multidisciplinary team discussions.

A wide range of software platforms and printing technologies were employed across studies. The PLUTO system [48, 49, 59] and 3D Slicer [54] were recurrently used for segmentation and STL file generation, whereas open-source tools such as Horos, Meshmixer, Blender, and Ultimaker Cura were frequently integrated into low-cost workflows [50, 52, 54]. The dominant fabrication techniques were Fused Deposition Modeling (FDM) or Fused Filament Fabrication (FFF) [50, 52, 54, 55, 58], followed by Stereolithography (SLA) [57] and Selective Laser Sintering (SLS) [58].

Material selection demonstrated notable heterogeneity. PLA was the most widely used polymer [50, 52, 54, 58], often combined with silicone to replicate the texture of hepatic parenchyma [52, 54, 58]. Other materials included rigid acrylic resin [48, 49, 59], photosensitive resin [57], transparent polyurethane rubber (TPUR) [28], and acrylonitrile butadiene styrene (ABS) [51]. The production cost of 3D-printed liver models ranged broadly, from as low as $15 [55] or €120 [52] to approximately €950–€1200 for high-fidelity, multi-material models [53]. Similarly, the manufacturing time varied from 6 h [55] to 72 h [50], with most reports indicating a total workflow duration of one to five days per model [54].

## Discussion

In our single-center study, we demonstrated that 3D-printed patient-specific models can be effectively used for preoperative planning in complex HPB surgeries evaluating their impact from both clinical and educational perspectives. Furthermore, an extensive cost analysis confirmed that “in-house 3D model production” is financially attainable for a surgical department.

### Anatomical fidelity and clinical utility

The 3D models produced were highly accurate representations of patient anatomy, as supported by the lack of intraoperative surprises or model inaccuracies noted [61–63]. This high fidelity is attributable to meticulous imaging segmentation and the use of dual-modality data (CT and MRI), which improves reproducibility and feature stability [16, 64], as well as the intrinsic resolution capabilities of modern printers [26, 39, 65–68].

High anatomical accuracy of 3D-printed models has been reported in several studies[33, 69–72]. By allowing surgeons to see and touch a replica of the patient’s organs and tumor, the models provided an intuitive understanding that complemented traditional imaging. In all 3 cases, surgeons found that the model—combined with preoperative CT and/or MRI imaging and IOUS—enhanced their 3D mind map of the operative field.

Furthermore, the models could influence surgical decision-making. For instance, in one case the presence of the model prompted a proactive plan for vascular repair, which likely contributed to a safer resection. In another, the model enabled preservation of an accessory artery, illustrating how patient-specific models can facilitate more conservative, anatomically informed surgeries.

These qualitative benefits align with literature reports of 3D models changing or refining surgical strategies [54, 73, 74]. It has been documented that 3D models can lead surgeons to adjust resection extent or approach in a significant percentage of cases [54, 73]. Our finding that R0 resections were achieved in all cases involving tumors (with margins tailored to the tumor anatomy seen in the model) is encouraging, though our sample is limited.

We cannot claim based solely on this series that the models improved oncologic outcomes, but they certainly provided reassurance and clarity in achieving margin-negative resections. Prior studies have suggested that using 3D models in liver and pancreas surgery may reduce positive margin rates and operative time, as well as intraoperative blood loss [75–77].

The 3D-printed models did not reveal entirely new anatomical information beyond imaging, but they synthesized that information into a more understandable form [2, 78]. Experienced surgeons are trained to interpret CT and MRI, but certain spatial relationships (such as the exact curvature and proximity of a vessel encircling a tumor) are undoubtedly easier to grasp with a tangible 3D model [79–81].

For example, the SMA and SMV and their contact with the pancreatic uncinate tumor were apparent on axial CT, but the 3D model allowed the team to appreciate the circumferential nature of involvement and plan the dissection. In the literature, 3D virtual reconstructions on screen provide some of this understanding, but physical models offer depth perception and the ability to manually simulate surgical maneuvers, which can be invaluable [2].

Our study underscores that 3D models serve as an excellent adjunct to conventional imaging. Surgeons still rely on imaging for diagnostic details and initial assessments; the model then acts as a supplementary tool that can confirm findings and improve confidence in the plan [62, 67].

In the era of precision medicine, the standardization of acquisition protocols and the accurate review of radiological images allow a more efficient multidisciplinary discussion and the selection of a personalized treatment strategy involving surgery, radiotherapy, local treatments, or chemotherapy [82–84].

Furthermore, although our study was not designed to measure clinical outcomes (operative time, complication rates, patient recovery), the goal of improved preoperative planning is to translate into better surgical outcomes. Literature suggests potential improvements. Witowski et al. [54] observed reductions in operative time and blood loss using 3D liver models for planning hepatectomies.

Similarly, a recent case-matched study found that 3D model-guided liver resections had a higher likelihood of achieving an R0 resection and preserving more healthy parenchyma compared to controls [85]: These benefits could be linked to more precise knowledge of patient anatomy and tumor extension preoperatively.

Moreover, in our anecdotal experience, 3D model visualization may help reduce operative time by clarifying intraoperative anatomy or by enabling the team to make and simulate ad *hoc* decisions.

An AI-based model can even allow the integration of the most recent post-processing technologies concerning body composition and anatomical detail to complement the preoperative evaluation with crucial information on parenchymal structure or liver function [86, 87].

A crucial point is the potential benefit of reducing operating room team stress: The 3D model could enhance team communication thanks to case illustrations during preoperative briefing.

Some authors have also described using 3D models for patient education—showing the patient their own tumor and planned resection on a model to improve informed consent and satisfaction [88–90]. While we did not formally measure patient perceptions in this study, we anecdotally did use the models to explain the surgery to the patients and families. This suggests an additional “softer” benefit of 3D printing: improved patient engagement and possibly reduced anxiety, as they can literally see what the surgery entails.

Furthermore, the adoptions of 3D-printed models are particularly relevant in the era of minimally invasive surgery which represents a suitable approach for liver and pancreatic surgery [91, 92] and where tactile feedback is limited, while precise anatomical orientation is essential.

### Learning outcomes

The randomized survey component of our study provided clear evidence that 3D-printed models significantly enhance the learning of complex anatomy. Students who had access to a 3D model outperformed those who did not, particularly in understanding spatial relationships.

The large improvement in the 3D Anatomy domain (by over 2 points on average, with *p* < 0.001) indicates a strong effect size of the educational intervention.

This finding is consistent with previous educational research. For instance, a systematic review by Ye et al*.* [33] found that 3D-printed models improved anatomy examination scores and spatial understanding for medical trainees, compared to traditional methods. Likewise, Hojo et al. [93] demonstrated in a randomized trial that surgical trainees who practiced on 3D pelvic models had better performance in a simulated lymph node dissection than those who did not.

Our study adds to this body of evidence by focusing on HPB anatomy—a challenging field—and by using a randomized design at the undergraduate medical education level.

The improvement in the experimental group’s scores suggests that even a brief exposure to the 3D model provided an immediate learning benefit. We also observed that model exposure did not significantly change performance on pure factual knowledge questions, implying that 3D models specifically augment the spatial cognitive domain. Indeed, the value of these tools lies in conveying 3D structure and relationships, which are hard to fully capture in words or flat images. Traditional lectures and textbooks remain important for factual and conceptual knowledge, but incorporating 3D-printed models or even 3D digital models could fill the gap in spatial learning [94].

From a curriculum standpoint, our results support the integration of physical anatomical models in teaching complex anatomy to improve student comprehension and potentially long-term retention.

These findings could be crucial during surgical training, especially in the era of telemedicine [95]: a junior surgeon could use the model to practice the surgical approach or identify anatomical landmarks before assisting or performing the case, as reported in previous studies [96–99].

Future surgical education should increasingly integrate 3D-printed models and even augmented reality overlays of patient anatomy as standard tools accelerating the learning curve especially in low-, medium-volume centers [100, 101] also building preoperative high-fidelity simulators as reported in neurosurgery and orthopedics experiences [102–104].

### Cost and feasibility considerations

One of the barriers to widespread adoption of 3D printing in hospitals has been the presumed cost and need for technical expertise.

Our detailed cost analysis demonstrates that in-house production of basic anatomical models can be done at low cost. Roughly €50–€60 per model in consumables is trivial in the context of overall surgical care costs (for comparison, a single advanced imaging scan or an operative instrument can cost many times that). Even considering staff time, the cost remains justifiable, especially if models are only made for select complex cases.

In our setting, this was facilitated by having a collaborative team of surgeons and bioengineers. Other centers have reported similar workflows where either surgical residents or medical illustrators handle segmentation and 3D model preparation [105, 106].

Streamlining this process will improve feasibility: For instance, advances in automated segmentation using AI may drastically cut down the time needed to generate a printable model.

Several commercial services provide 3D-printed models from uploaded imaging, but they often charge in the thousands of euros/dollars per model, resulting in not cost-effective tools for routine use [103, 107].

In our experience, the major “cost” is time—specifically print duration. A 30-h print means one must plan ahead, so the model is ready before surgery. In practice, this was manageable by starting prints well in advance and printing continuously (our printer ran overnight and was monitored for errors). As printer technology improves, faster printers or multi-head printers could cut print times significantly.

Thus, while some logistical planning is required, we did not find any prohibitive hurdles in implementing a small 3D printing program for surgical planning. The learning curve for segmentation software is moderate, but once a protocol is established, it can be taught to tech-savvy members of the surgical team.

Several free or low-cost software options exist, and many hospitals already have 3D laboratories or experience in printing for orthopedic implants or other uses, which can be extended to anatomical modeling [108, 109].

However, it must be acknowledged that not all surgical centers may have the technological infrastructure or financial resources to implement such tools routinely. It is therefore evident that the outcomes of complex surgical procedures may not solely depend on the surgeon’s technical expertise but are also influenced by institutional resource availability and access to advanced technological support [110, 111].

### Limitations

#### Our study has several limitations

The sample size of three clinical cases is small, and our observations of clinical utility are descriptive. We did not have a matched control group of patients planned without 3D models to directly compare surgical outcomes. A larger series or controlled trial could more rigorously evaluate metrics like operative time, intraoperative blood loss, or complication rates with vs. without model planning.

Secondly, the production process in our study was resource-intensive in terms of personnel—it relied on dedicated individuals with interest in 3D printing. Not all surgical centers may have immediate access to such expertise, which could hinder widespread adoption. However, this is mitigated by the rapidly increasing accessibility of 3D printing technology and knowledge sharing.

Another limitation is that our cost analysis did not account for personnel time in monetary terms (since this was a research context). In a real-world scenario, the time surgeons or staff spend on model creation could be seen as a cost. That said, this could be integrated into existing workflows (e.g., radiology could incorporate segmentation as part of advanced imaging services).

## Conclusions

3D-printed anatomical models were successfully implemented for preoperative planning in a series of complex HPB surgeries, and they proved to be highly accurate and useful. The availability of a patient-specific 3D model allowed surgeons to better visualize tumor anatomy and its relationships with critical structures, leading to refined surgical plans and increased confidence in the operating room.

Furthermore, in an educational context, exposure to 3D-printed models significantly enhanced medical students’ understanding of complex 3D anatomy.

Future studies with larger cohorts and quantitative outcome measures are warranted to further define the impact of 3D-printed models on surgical results and to solidify their role in surgical pedagogy.

## Data Availability

The raw data supporting the conclusions of this article will be made available by the authors, with undue reservation.
